# Elucidation of Biochemical Pathways Underlying VOCs Production in A549 Cells

**DOI:** 10.3389/fmolb.2020.00116

**Published:** 2020-06-30

**Authors:** Takeshi Furuhashi, Ryuga Ishii, Haruka Onishi, Shigenori Ota

**Affiliations:** ^1^Anicom Specialty Medical Institute Inc., Tokyo, Japan; ^2^GL Sciences Inc., Saitama, Japan

**Keywords:** cellular volatile, VOC (volatile organic compounds), lipid peroxidation, GC-MS, lung cell

## Abstract

Cellular volatile organic compounds (VOCs) are unique compounds whose metabolic pathways remain enigmatic. To elucidate their metabolism, we investigated the VOCs of lung cancer A549 and 2 non-cancer lung cells (HLB; HBEpC). Neutral sugars and lactate in the medium were measured by colorimetric assay. VOCs were enriched by monotrap and profiled by GC-MS. To investigate the enzymes that change VOC metabolism in cells, we conducted ALDH activity assays and qPCR. ROS (reactive oxygen species) assays were conducted to assess oxidation stress. The colorimetric assay showed that especially A549 and HLB took up sugars from the medium and rapidly secreted lactate into the medium. The VOC profile (GC-MS) revealed a *trans*-2-hexenol increase, especially in A549 lung cancer cells. This is a novel lipid peroxidation product from animal cells. Based on the absolute quantification data, *trans*-2-hexenol increased in parallel with number of A549 cancer cells incubated. The qPCR data implies that ADH1c potentially plays an important role in the conversion into *trans*-2-hexenol.

## Introduction

The potential relationship between VOCs (volatile organic compounds) emitted by the human body and malignant diseases (e.g., cancer) has been investigated since Hippocrates' time (Di Francesco et al., 2005). Recent technological advances have provided new knowledge about the metabolic changes related to VOC production in diseases, and VOC profiles enable comparison between normal and disease conditions (Hakim et al., 2012).

Nonetheless, a gap remains between disease detection and volatile compound analysis. One reason is that the relationship between pathology and the metabolic pathways producing VOC is not fully understood. The mechanism of biological VOC synthesis in cells is enigmatic because many studies on such metabolites are restricted to bacteria, algae, and fungi (Ruffing, 2013). Moreover, the enzymes associated with profiled cell VOCs have not always been identified (e.g., hydrocarbons, alcohols, alkenes). The goal of our study is to elucidate the VOC pathway which can potentially be marker for cancer. VOC analysis by GC-MS profiles (Buszewski et al., 2012) is certainly useful for assessing VOCs as biomarkers, but there is some room for improvement in VOC sampling.

In fact, direct gas injection or headspace analysis (Snow and Slack, 2002) normally show lower VOC values than methods using fiber-based VOC sampling and enrichment. Two types of fiber-based sampling are currently available. One is a fiber attached to a syringe which is exposed to gas by incubation (typical for SPME; solid phase micro extraction). The other is a naked fiber shape (typical for monotrap). In the case of SPME, only the fiber surface can retain VOCs, whereas in monotrap the VOCs are retained both by the surface and the inner porous part. This opens the door for improving the intensity of VOC detection as well as for introducing disposable VOC-capturing materials.

Monotrap is a disposable sorptive media based on the large surface area of silica monolith. It has been applied in biological research and can be preserved in the sample bottle prior to measurement (Jang et al., 2011; Ma et al., 2013). Nonetheless, despite the large surface for capturing VOCs, monotrap has been used solely for static sampling. This means that improving the preconcentration technique (e.g., NTME; needle trap extraction and ITEX; in tube extraction) requires shorter incubation time but generates better intensity (Mochalski et al., 2018) and should be applicable to single cell studies as well (Serasanambati et al., 2019). For this reason, we developed an active sampling approach for monotrap-based sample preparation which is suitable for cellular VOC analysis.

Moreover, solely a VOC profile is insufficient to elucidate a synthetic pathway. To date, a VOC profile has not been combined with any enzyme assay. In this study, we conducted enzymatic assays to find key enzymes related to marker VOC production.

## Materials and Methods

In this study, we used A549 (human lung adenocarcinoma), human lung fibroblasts (HLB) cells, and human bronchial epithelial cells (HBEpC) as primary cells. This is because lung cancer is one of the most lethal cancers and also because some VOC analyses have already been reported (de Lacy Costello et al., 2014; Lemjabbar-Alaoui et al., 2015). Cell cultures are normally done in 2-dimensional (2D) form, but this sometimes does not reflect actual physiology (Kapałczynska et al., 2018). Accordingly, we compare flasks with non-adherent plates, which can mimic 3D culture systems.

Based on the VOC profile results, we focus on *trans*-2-hexenol metabolism. ALDH (aldehyde dehydrogenase) assays were applied to assess the conversion of aldehyde groups into carboxylic acid groups. To investigate the speed of sugar uptake (as the main energy source) and to evaluate the glycolysis pathway, we conducted phenol sulfuric acid assays for the medium assay to determine sugar consumption from the medium. Lactate secreted into the medium was calculated using a lactate assay kit; this reflects the balance of reduction and oxidation inside the cell. qPCR (quantitative polymerase chain reaction) was applied to determine whether certain enzymes (i.e., generation of *trans*-2-hexenol) are related to the VOC profile or not. ROS (reactive oxygen species) assays were done to assess whether cells are under oxidation stress.

### Cell Culture Condition

A549 cells and human lung fibroblasts (HLB) cells were purchased from the American Type Culture Collection. Immortalization was done to HLB. A549 cells were originally isolated from a lung carcinoma of a 58-y-old man, showed epithelial morphology and grew adherent. All cells were grown in DMEM (Dulbecco's modified eagle medium) high-glucose culture medium containing sodium pyruvate (110 mg/L) supplemented with 10% FCS (fetal bovine serum), penicillin (100,000 units/L), streptomycin (100 mg/L), and L-glutamine (293 mg/L). Human bronchial epithelial cells (HBEpC) are primary cells (PromoCell GmbH) isolated from the mucosa of the main bronchi of a 42-y-old male Caucasian. The cells were cultivated in Airway Epithelial Cell Growth Medium (PromoCell GmbH) supplemented with the Airway Epithelial Cell Growth Medium Supplement Pack (PromoCell GmbH) according to the manufacturer's instructions. A T75 cell culture flask (250 mL, 75 cm^2^) was used for the culture. For 2-dimensional (2D) and 3-dimensional (3D) cell culture, flasks with a red screw cap (CellStar 618175) and cell-repellent surface flasks with white screw caps (CellStar 658985), respectively, were used. Cultured cells were observed, and photographs were taken using an OLYMPUS IX71 microscope (OLYMPUS) and software AdvanView 3.7 (AdvanVision Co, Ltd).

For all experiments, cells were cultivated under standard conditions at 37°C in a humidified atmosphere with 92.5% air/7.5% CO_2_. All cells (1 × 10^6^) were inoculated in 20 mL phenol red-free DMEM high-glucose medium (supplements: 5% FCS, 100,000 units/L penicillin, 100 mg/L streptomycin, 293 mg/L L-glutamine, and 110 mg/L sodium pyruvate). After Days 3 and 4, cells were collected by digestion with 0.25% Trypsin-EDTA (Thermo Fisher) for 5 min at 37°C. After trypsinization, medium (DMEM containing 10% FBS) was added and mixed by pipetting cells. Cells were collected by centrifugation and resuspended in fresh medium. Cell counts were performed on a Cell Counter model R1 (OLYMPUS, USA).

The FCS concentration in DMEM during the experiment was lowered to 5% to reduce the high background of VOCs in the analyzed headspace. Cells were grown in these conditions for 96 h, up to a confluence of 50–60% (around 1.0 × 10^6^ cells/flask). To quantify sugar and lactate in the medium, Days 1, 2, 3, and 4 were sampled. The same days were sampled for the cellular ALDH activity assay and qPCR. Cells were transferred into a new flask 4 days after inoculation. Physiologically, Day 0 cells were the same as Day 4 cells. Considering the accumulation of VOCs inside the cell culture flasks, Days 0, 3, and 4 samples were collected for VOC analysis.

### Phenol-Sulfuric Acid Assay

Neutral sugars as cellular energy sources were quantified by the phenol-sulfuric acid assay (modified from a previous study; (DuBois et al., 1956)). At each time point, 5 μL culture medium was taken from the flask and transferred into a 2 mL Eppendorf tube. We then added 195 μL deionized water and 80% 5 μL phenol solution. Thereafter, 500 μL of concentrated sulfuric acid was added by titration (on ice). The solution was mixed by inverting the tube and incubated for 25 min at room temperature. We then transferred 150 μL into 96-well plates and measured absorbency at 490 nm using Enspire (Perkin-Elmer, USA). Each measurement was biologically replicated 4 times. Quantification involved using a linear calibration curve with glucose standard solution (0.5–100 μg).

### Lactate Assay

A Lactic acid Assay Kit (10139084035, R-Biopharm AG, Germany) was used to quantify lactate in the medium. At each time point, 1 μL culture medium was taken from the flask and 99 μL deionized water was added. The solution was transferred to 96-well plates, and 100 μL glycylglycine buffer (440 mg/30 mL, pH 10) and 20 μL NAD (nicotinamide adenine dinucleotide) solution (210 mg/6 mL) were added. After that, 2 μL glutamate-pyruvate transaminase suspension (1,100 U) was added. The solution was incubated at room temperature for 5 min. Absorbency at 340 nm was measured by Enspire (A_1_). Then, 2 μL lactate dehydrogenase solution (3,800 U) was added and incubated at room temperature for 30 min. Absorbency at 340 nm was measured by Enspire (A_2_). The lactate concentration was calculated as A_2_-A_1_. Each measurement was biologically replicated 4 times. A linear calibration curve with lactate standard solution (10–2,000 ng) was used for quantification.

### Monotrap VOC Enrichment-Thermal Desorption (TD)

In monotrap, the mass transfer can be very slow due to a thicker membrane than in SPME. This requires an alternative agitation/convection strategy especially designed for monotrap. In this study, we tested pumping with monotrap, which is active sampling to enrich VOC by continuously pumping air. This enables capturing large volumes of VOCs within 30 min. Graphite and C18 type monotrap were used for this study. At each incubation period (Days 0, 3, and 4), VOCs were collected with a special penetrating vent cap (modified after a previous study; Schallschmidt et al., 2015). Each measurement was done in triplicate. For VOC quantification with different numbers of cells and to determine a limit of detection, a calibration curve was prepared using reference compounds. Three VOCs (i.e., *trans*-2-hexenol, tetradecane, isobutyrate) were chosen from VOC GC-MS profile data. Reference compounds for absolute quantification were purchased from SIGMA and TCI company (*trans*-2-hexenol, SIGMA 132667; tetradecane, TCI T0079; and isobutyrate, SIGMA l1754). For preparing the calibration curve, reference compounds were added into 10 mL deionized water. The solution was put into the cell culture flask, and subsequently the volatilized compounds were trapped by monotrap with 30 min pumping and measured by GC-MS. For comparison, 1 × 10^6^, 2 × 10^6^ A549 cells and cell-less media as negative control were compared. The incubation period was Days 0 and 3.

The experimental set-up used for the cell culture headspace analysis is shown in Figure S1. The control medium was obtained by incubating DMEM culture medium in the same conditions as the cell samples, but without seeded cells.

A syringe was connected to the cell culture flask by a penetrating screw cap hole. Two monotraps, graphite type (MonoTrap GC TD, Cat.No. 1050, GL Sciences, Tokyo) and C18 type (MonoTrap RSC18 TD, Cat.No. 1050-73201, GL Sciences, Tokyo) were put into the syringe (TERMO 2.5 mL, SS-02SZ). Pumping (~1,500 times up and down in total) was repeated for 30 min. After 30 min pumping, monotraps (both graphite type and C18 type) were removed and put tandemly into a Handy-TD glass liner (MonoTrap TD Liner for Handy-TD Cat.No. 1003-75005). As we used a T75 flask (270 mL volume containing 10 mL aqueous solution) and pumped using a 2.5 mL syringe, the total head space volume was 270 + 2.5 – 10 = 262.5 mL.

In preliminary experiments, we determined that 20–30 min was sufficient for the monotrap to capture gases. The liner was inserted into the Handy-TD.

### GC-MS Conditions

GC-MS measurements were carried out on a single quadrupole mass spectrometer (5977B-MSD; Agilent Technologies, Santa Clara, CA, USA) equipped with 7890B GC (Agilent Technologies, Santa Clara, CA, USA) and Handy-TD265 (GL science, Iruma city, Japan). The conditions were modified from a previous study (Furuhashi et al., 2018). The initial Handy-TD condition was a constant purging flow of 5 mL/min of helium gas at 40°C for 0.1 min. Predesorb was 70 kPa. For desorb, the ramp rate was 45°C/s, with 1.5 min holding at 250°C. GC-MS measurement was started directly after Handy-TD desorb.

The temperature of the liner was 230°C. The GC column helium gas flow was set at 1 mL/min constant rate. DB-Wax UI 30 m, 0.25 mm, 0.25 μm (122-7032 UI, Agilent, USA) was used as a GC column. The oven temperature gradient for the samples was as follows. After a 5 min, 50°C isothermic period, the oven was programmed to rise to 150°C at a rate of 5°C min^−1^, then rise to 260°C at a rate of 40°C min^−1^, held at 260°C for 1 min. The temperature of both the GC-MS ion source and transfer line was set at 250°C. The scan range was between *m*/*z* 30 and 600. For quantification, we calculated the peak area of a conventional 70 eV EI mode (Extractor ion source; Agilent Technologies, Santa Clara, CA, USA) extracted ion chromatogram using software (Mass Hunter; Agilent Technologies, Santa Clara, CA, USA). The chosen *m*/*z* and retention time for quantification are described in Table S1. The sample was measured with split mode. Cellular VOCs were measured with a split ratio of 3. Between batch measurements, C17:0 FAME was measured to check the intensity between different batches as well as the retention time shift.

### qPCR Condition

At each time point, culture medium was removed by centrifuging (3,000 g for 5 min). Cells were washed with PBS (phosphate-buffered saline). For 2D culture cells, cells were detached from the flask by scratching with a scraper. The cell suspension was transferred into 1 mL Eppendorf tubes and centrifuged at 200 g for 5 min. The supernatant was aspirated.

Total RNA was extracted from cells using TRIzol Reagent (Cat: 15596026, Thermo Fisher Scientific, USA) according to the manufacturer's protocol. mRNA was reverse transcribed using an oligo (dT) primer and SuperScript III Reverse Transcriptase (Cat: 18080400, Invitrogen, USA) (Ishii et al., 2012). For the thermal cycle reaction, the cDNA template was amplified by the thermal cycler LightCycler 96 System (Roche Diagnostics, USA) using the FastStart Universal SYBR Green Master (Rox) (Cat: 04 913 914 001, Roche Diagnostics, USA) under the following reaction conditions: 40 cycles of PCR (95°C for 10 s, and 60°C for 1 min) after an initial denaturation (95°C for 10 min). Fluorescence was monitored during every PCR cycle at the annealing step. The authenticity and size of the PCR products were confirmed using a melting curve analysis with the software LightCycler 96 System version SW1.1 (Roche Diagnostics, USA). mRNA levels were normalized using Glyceraldehyde-3-phosphate dehydrogenase (GAPDH) as a housekeeping gene. Primer sequences are provided in Table S2.

### ALDH Assay

The ALDH Assay Kit (K731-100, BioVision, USA) was used to quantify cellular ALDH activity. At each time point, culture medium was removed by centrifuging (3000 g for 5 min). Cells were washed with PBS. For 2D culture cells, cells were detached from the flask with a scraper. The cell suspension was transferred into 1 mL Eppendorf tubes and centrifuged at 200 g for 5 min. The supernatant was aspirated and 200 μL ALDH assay buffer was added. The cell suspension was homogenized by vortex and incubated on ice for 10 min. The suspension was centrifuged at 20,000 g for 3 min and the supernatant transferred to a new tube. The supernatant solution was incubated at room temperature for 10 min. Absorbency at 450 nm was measured by Enspire at 0, 20, 40, 60 min. As enzymatic activity corresponded to NADH generation during the reaction time, the slope of absorbency at 450 nm and time (X axis) was calculated. The obtained slope was normalized with cell number (1 × 10^5^ cells) and then multiplied times 10^4^. Each measurement was biologically replicated 4 times. The cell number for the normalization of ALDH activity was determined with an OLYMPUS cell counter model R1 (OLYMPUS).

### ROS Assay

The Cellular ROS assay kit (Red) (ab186027; Abcam, Cambridge, UK) was used. The protocol was modified from the ab186027 protocol. 2D or 3D cultured cells (HLB/HBEpC/A549) were prepared (2D or 3D culture for 1–4 days incubation). After trypsin treatment, cells were washed with PBS two times. The cell suspension was prepared as 5 × 10^4^ in 100 μL PBS. After pipetting the cell suspension 3 times, 50 μL suspension was transferred into a microplate (655077, 96 well, F-bottom, black, Fluotrac; Greiner Bio-one, Germany). We added 50 μL ROS red working solution (2 μL ROS red stock solution in 1 mL ROS assay buffer) into cell suspension. We then incubated the microplate at 37°C with 5% CO_2_ for 1 h. After incubation, fluorescence was measured as Ext 520 nm Ems 605 nm using Enspire (Perkin-Elmer, USA). Each measurement was biologically replicated 4 times.

## Results and Discussion

**Figure 1:**
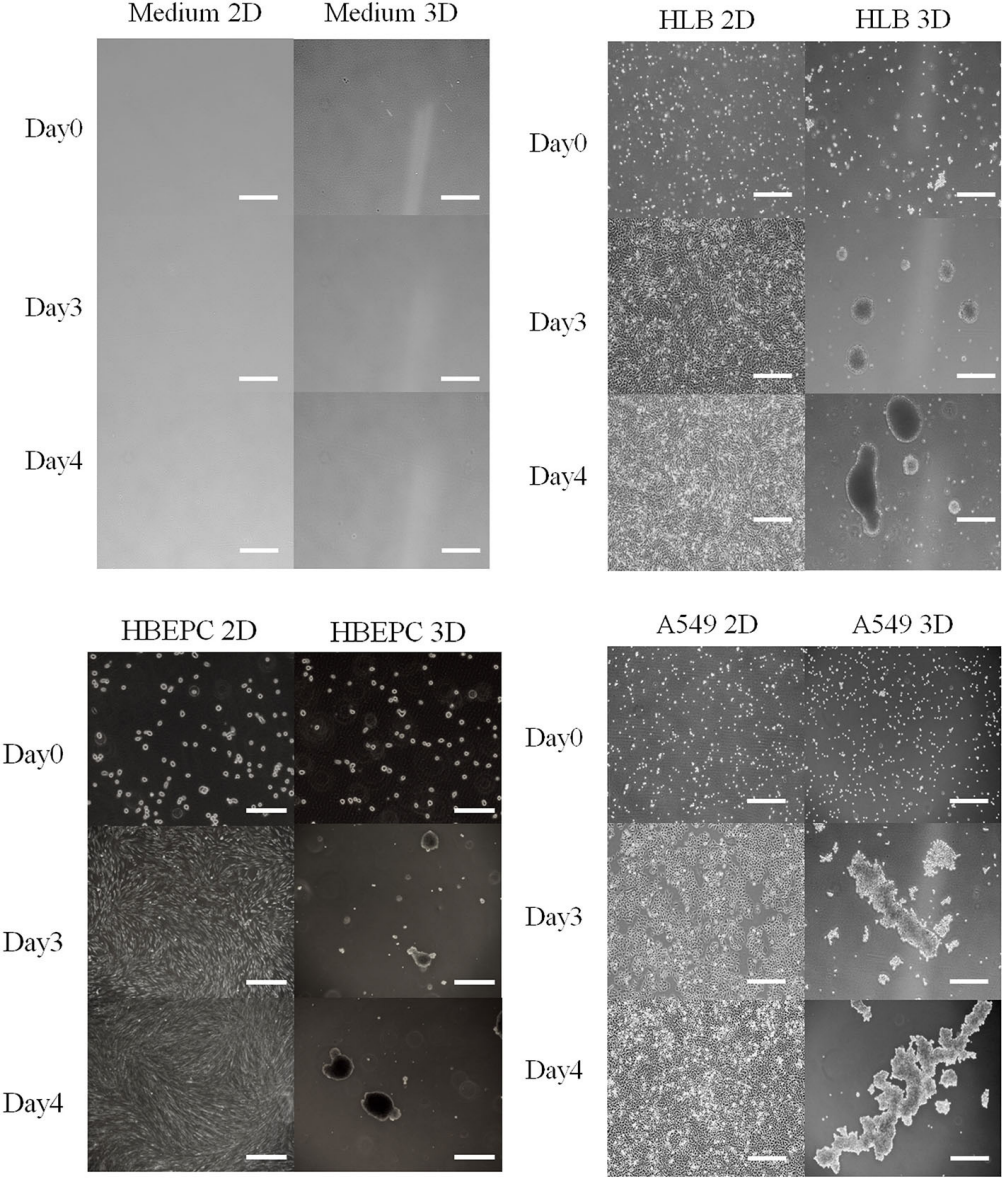
Photos of medium as negative control, HLB (Human lung fibroblasts cells), HBEpC (Human bronchial epithelial cells as primary cells), and A549 (Human lung adenocarcinoma) from Days 0 to 4 in both 2D and 3D culture. All cell types in 2D culture spread across the bottom of the flask, whereas those in 3D culture aggregated. Photos were taken at 0, 3, 4 days after inoculation. Scale bar: 500 μm except for HBEpC at Day 0 (200 μm).

**Figure 2:**
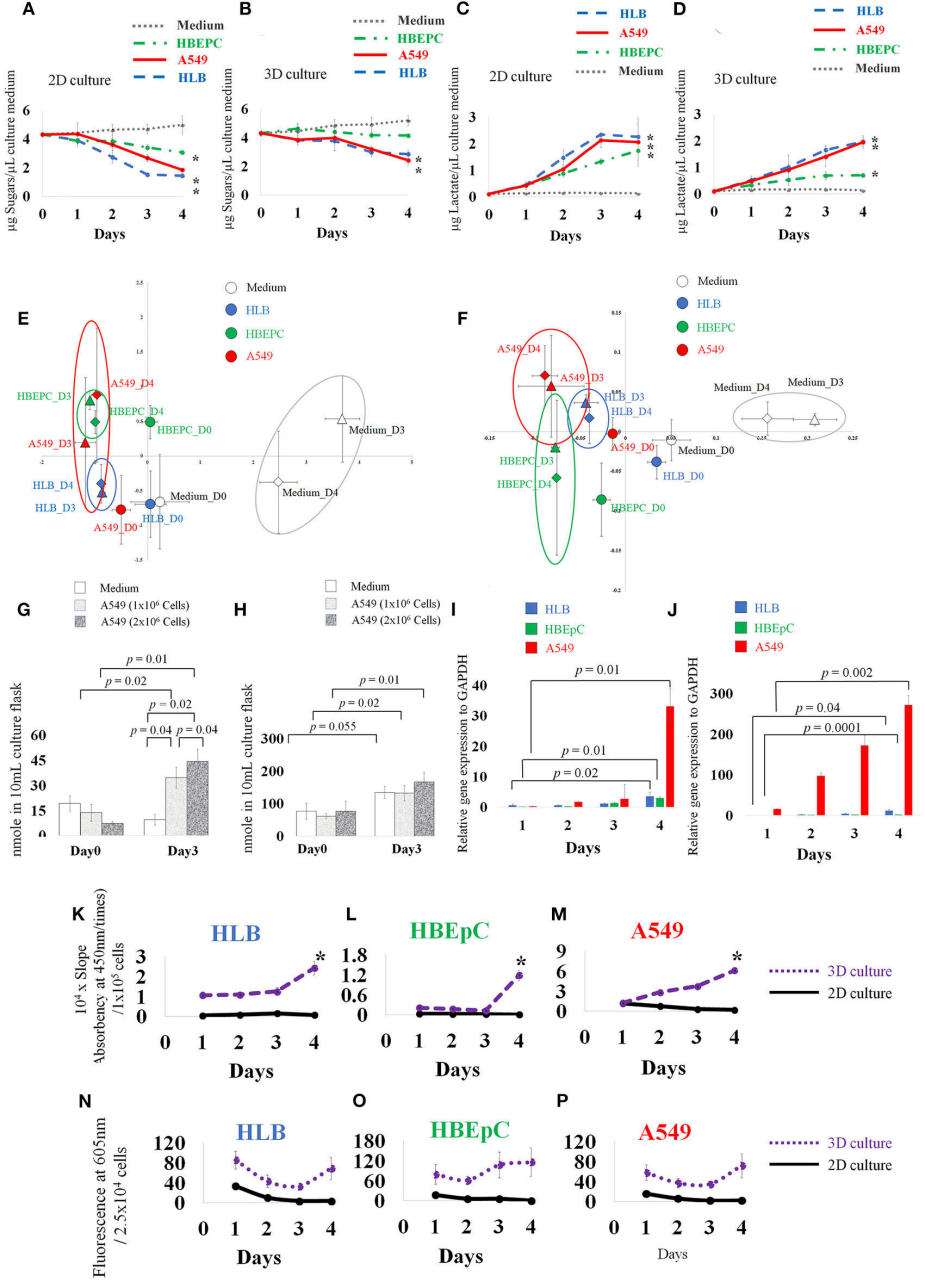
Concentration of medium sugars **(A,B)**, and lactate **(C,D)**. Principle component analysis (PCA) based on 30 VOC GC-MS data of 2D and 3D cultures **(E,F)**, and absolute quantification of *trans*-2-hexenol and isobutyrate in 2D-cultured cell-less media and 1 × 10^6^ and 2 × 10^6^ A549 cells (human lung adenocarcinoma) A549 cells **(G,H)**. qPCR data **(I,J)**, ALDH (aldehyde dehydrogenase) activity assay over time **(K–M)**, and ROS data **(N–P)**. HLB (Human lung fibroblasts cells), HBEpC (Human bronchial epithelial cells as primary cells), and A549 (human lung adenocarcinoma) were compared. Note different scales on y-axis. **(A)** Neutral sugar concentration in 2D-cultured medium. **(B)** Neutral sugar concentration in 3D-cultured medium; Y axis indicates μg Sugars/μL culture medium, error bars indicate standard deviation. **(C)** Lactate concentration in 2D-cultured medium. **(D)** Lactate concentration in 3D-cultured medium. Y axis indicates μg Lactate/μL culture medium, error bars indicate standard deviation. Medium without cells, HLB (Human lung fibroblasts cells), HBEpC (Human bronchial epithelial cells as primary cells), and A549 (Human lung adenocarcinoma) were compared. For the sugar and lactate assays, sampling was done at 0, 1, 2, 3, 4 days after inoculation. Experiments were biologically replicated four times. Asterisk indicates statistical significance (*p* < 0.05) between Day0 and Day4 of each cellular sample. **(A)** The sugar concentration in 2D culture medium fell on day 4, from 4.3 (±0.2) μg/μL medium to 1.4 (±0.1), 3.0 (±0.1), and 1.8 (±0.1) μg/μL medium in HLB, HBEpC, and A549, respectively. The corresponding *p*-values (0 day to 4 days after inoculation) were 0.0000008, 0.00005, and 0.002. **(B)** Sugar concentration in 3D cell cultures in HLB and A549: values 4 days after inoculation were 2.8 (±0.3) and 2.4 (±0.3) μg/μL medium, and *p*-values compared with 0 day were 0.0002 and 0.00006, respectively. **(C)** Lactate secretion into the medium of 2D-cultured cells, 4 days after inoculation, reached 2.2 (±0.4), 1.7 (±0.3), and 2.0 (±0.9) μg lactate/μL medium in HLB, HBEpC, and A549, respectively; corresponding *p*-values (0 day to 4 days after inoculation) 0.002, 0.002, and 0.01. **(D)** In 3D-cultured cells, the lactate concentrations increased in the medium 4 days after inoculation in HLB, HBEpC, and A549: 1.9 (±0.2), 0.6 (±0.05), and 1.9 (±0.1) μg lactate/μL medium, respectively; corresponding *p*-values (0day to 4days after inoculation) 0.0004, 0.000001, and 0.0001. In PCA, X and Y axis indicate PC1 and PC2, respectively **(E,F)**. Error bars: standard deviation of each group (*n* = 3). Each cell type after 3 days and 4 days incubation is circled. In absolute quantification graph **(G,H)**, Y axis indicates nmole VOC in flask. For quantification, extracted ion counting (EIC) of *trans*-2-hexenol fragment (*m/z* 82) and isobutyrate fragment (*m/z* 73) was used. Sampling was done at 0 and 3 days after inoculation. Experiments were biologically triplicated. Error bars: standard deviation. *p*-value showing statistical significance is listed. qPCR data **(I,J)** of ADH1c (alcohol dehydrogenase) in 2D- and 3D-cultured HLB, HBEpC and A549. Y axis indicates relative gene expression to GAPDH. In qPCR, sampling was done at 1, 2, 3, 4 days after inoculation. Experiments were done in triplicate. Error bars: standard deviation. *p*-value between Day1 and Day4 showed statistical significance. In the ALDH assay **(K–M)** and ROS assay **(N–P)**, experiments were biologically replicated four times. Error bars: standard deviation. Sampling was done at 1, 2, 3, 4 days after inoculation. Y axis in ALDH assay indicates 104 times multiplied slope (absorbency at 450 nm over time) which is normalized by cell number (105 cells). In the ROS analysis, Y axis indicates fluorescence at 605 nm/2.5 × 104 cells.

All 2D-cultured cells proliferated and spread on the bottom of the flask 3 days after inoculation, and they nearly overflowed 4 days after inoculation (Figure 1). The 3D-cultured cells proliferated with aggregation (Figure 1). Data obtained by the conventional phenol-sulfuric acid assay indicated that the neutral sugar concentration of all cell culture media gradually decreased over time in the 2D cell culture (Figure 2A). The tendency was similar in 3D cell cultures (Figure 2B) in HLB and A549. In the HBEpC cell culture medium, the neutral sugar concentration did not decrease significantly (*p*-value of 0–4 days after inoculation: 0.2). Both 2D and 3D culture medium without cells showed no decrease in medium sugar concentration.

Lactate secreted into the medium gradually increased with time in all 2D-cultured cells, (Figure 2C) and 3D-cultured cells, except that the lactate increase of HBEpC was moderate (Figure 2D). No change was recorded in 2D and 3D culture media without cells. Previous reports suggest that tumor cells under heterogeneous microenvironments can enter into a mutual relationship via lactate transport (Sonveaux et al., 2008). Our lactate assay data showed that both 2D- and 3D-cultured cancer cells secreted lactate into the medium, although the secretion speed in the latter was slower than in the former. Interestingly, compared with other cancer cells, the sugar uptake speeds from the media were slow only in 3D-cultured HBEpC, and the secretion of lactate into the media remained at a lower level. In general, rapid sugar uptake and lactate secretion were conspicuous in cancer cells (A549) and in normal cells that were immortalization treated (HLB), leading us to conclude that such characteristics are common in immortalized cells and are not cancer specific.

From the 81 VOC reference compounds list for this study (Table S1) - including hydrocarbons, aldehydes, alcohols, fatty acids, methyl esters, and aromatic groups −30 VOC peaks were detected in the flask based on the data obtained (Figures S2–S4). PCA (principle components analysis) based on these 30 VOCs (Figures 2E,F) showed that the VOC profiles of the medium were separated from those of cells both in 2D and 3D culture. After 3 days incubation, the difference between solely medium and cells began to increase. In 3D culture, the separation between cells was clearer than in 2D culture after 3 days incubation.

To investigate the VOCs contributing to the group separation in PCA, statistical analysis (*t*-test; *p* < 0.05) (Table S3) revealed an increase of benzaldehyde in the medium and decrease in cells (except A549 2D culture Day 4). This tendency is consistent with a previous study on mesenchymal stromal/stem Cellular VOCs (Klemenz et al., 2019). Tetradecane from all cellular samples increased with time. *trans*-2-hexenol conspicuously increased over time particularly in A549 cells in both 2D and 3D culture. The increase of *trans*-2-hexenol between medium and A549 at Day 4 was also significant (Tables S3, S4; Figure S4). Isobutyrate increased in A549 and HLB showed statistical significance in A549 3D culture and HLB 2D culture. A 3-methyl pentanoate increase was observed as an HBEpC-specific VOC but did not increase in other cells.

Based on the statistical analysis of VOC profile data, we focus on 10 VOCs (e.g., undecanal, tetradecane, nonanal, 2-ethyl-1-hexanol, isobutyrate, *trans*-2-hexenol, tridecane, toluene, acetate, undecane). These increased especially in 2D- or 3D-cultured A549 cancer cells at Day 4. Among these, undecanal, nonanal, 2-ethyl-1-hexanol, acetate, and tridecane showed statistical significance in A549 2D culture. In contrast, the increase was inconspicuous in the cell-less media. The increase of toluene and undecane in A549 Day 4 was statistically significant compared to cell-less media at Day 4, but these two compounds then decreased over time in the A549 incubation. *Trans*-2-hexenol, tetradecane, isobutyrate were positively correlated in cellular samples, showing that these VOCs increased together.

Accordingly, we selected *trans*-2-hexenol, tetradecane, and isobutyrate for quantification. The limits of detection were firstly determined as 5 nmole (*trans*-2-hexenol), 10 nmole (tetradecane), and 50 nmole (isobutyrate) in cell culture flasks with 10 mL aqueous solution. Linearity as a standard curve was valid between 5–500 nmole/10 mL (*trans*-2-hexenol), 20–1,000 nmole/10 mL (tetradecane), and 50–5,000 nmole/10 mL (isobutyrate) (Figure S5).

To confirm whether VOCs were derived from cancer cells or not, we compared 1 × 10^6^ and 2 × 10^6^ A549 cells. Both concentrations showed increased *trans*-2-hexenol. The difference was significant between Days 0 and 3 both in 1 × 10^6^ A549 and 2 × 10^6^ A549 cells (*p*-value 0.02 and 0.01, respectively) and and 2 × 10^6^ A549 cells (*p*-value 0.02 and 0.01, respectively) and between Day3 cell-less media and A549 cells (*p*-value 0.04 and 0.02 against 1 × 10^6^ A549 and 2 × 10^6^ A549 cells, respectively) (Figure 2G). The results show that the accumulation of *trans*-2-hexenol increased in parallel with the number of cells incubated (*p*-value between 1 × 10^6^ and 2 × 10^6^ A549 cells at Day 3 was 0.04). Our data show that about 20 nmole *trans*-2-hexenol/1 × 10^6^ A549 cells was secreted and accumulated in 3 days. In contrast, *trans*-2-hexenol was not accumulated in cell-less media. Isobutyrate increased significantly from Day 0 to Day 3 in A549 cells (*p*-values between Day 0 and Day 3 were 0.02 and 0.01 for 1 × 10^6^ and 2 × 10^6^ A549 cells, respectively) (Figure 2H). In cell-less media, isobutyrate also increased from Day 0 to Day 3 (*p*-value 0.055). For tetradecane, the detected EIC (extracted ion counting) peaks in the samples were below the limit of quantification. Based on the quantification results, *trans*-2-hexenol was chosen as the focus for further investigation.

We also observed a *trans*-2-hexenol increase in other cancer cells (Pancreas cell, PaCa2; and Neural cancer cell, YKG1) (unpublished data), indicating that *trans*-2-hexenol might be a commonly secreted VOC in many cancer cells. HLB, i.e., normal cells genetically modified as an immortalization treatment, took up sugars and secreted lactate to the same degree as cancer cells in both 2D and 3D cultures. At the same time, *trans*-2-hexenol did not increase in HLB, indicating that glucose uptake and proliferation alone do not lead to VOC production.

In considering the biosynthesis of *trans*-2-hexenol, lipid peroxidation and ROS are two important issues to understand. Cancer cells are typically heterogeneous and derived from normal cells by accumulating mutations (Aktipis et al., 2015). Such mutations lead to excess ROS inside the cells, and radical species react with cellular metabolites and genes. For instance, an ROS increase in cells leads to membrane degradation followed by lipid peroxidation. Lipid peroxidation, for example, generates toxic compounds such as aldehyde (Ayala et al., 2014). Aldehydes are highly reactive chemical substances whose internal content cells need to reduce.

There are several pathways to convert aldehyde into other compounds (i.e., alcohol, carboxylic acid and hydrocarbon). In the metabolic pathway of mammals, a conversion of aldehydes into hydrocarbons is unlikely because there are no reports that mammalian cells possess such an enzyme. Accordingly, aldehyde conversion probably mainly involves conversion into alcohol and/or carboxylic acid. Regarding genes related to *trans*-2-hexenol production, no information is available about AAR (Acyl ACP reductase) and ADC (Aldehyde decarbonylase) in the human genome. We therefore focused on enzymes that can generate alcohol, i.e., FAR (Fatty acyl-CoA reductase) and ADH (Alcohol dehydrogenase) or ALR (Aldehyde reductase).

Aldehyde conversion into alcohol by ADH (alcohol dehydrogenase = aldehyde reductase) is a reversible chemical reaction, whereas aldehyde oxidation into carboxylic acid is irreversible (Rizzo, 2014). A previous study on rat ADH reported that class I (i.e., ADH1) actively reduces 2-hexenal *in vitro* (Boleda et al., 1993). This suggests that mammalian cells can reduce aldehyde into alcohol and implies that ADH1 is related with the *trans*-2-hexenol increase.

As *trans*-2-hexenol has not been reported in previous lung cell studies (Filipiak et al., 2010, 2016), we further investigated enzymes to confirm this VOC production. From qPCR data, ADH1c of all cells increased 4 days after inoculation in both 2D and 3D culture, and it increased conspicuously especially in A549 (Figures 2I,J). The relative expression level to GAPDH was also much lower than that of A549. The mean values of relative expression to GAPDH 4 days after inoculation were 2.95 and 1.8 in 2D and 3D cultures, respectively. At the same time, the respective values of A549 were 33 and 271. Other ADHs (ADH1A, 1B, ADH3B1, ADH5, ADH9A1) showed no clear upregulation over time in either 2D or 3D cultures (unpublished data). We also examined other enzymes such as ALR2 and FAR1, which are related to alcohol production in cells (Figure S6), but there were no significant differences over time or according to cell type (HBEpC and A549). Apparently, ALR2 and FAR1 were not related to alcohol production, and *trans*-2-hexenol is produced mainly by ADH class I; moreover, *trans*-2-hexenol does not appear to be derived from fatty acyl CoA (Figure S7).

Other strategies are available to remove aldehydes, for instance a conversion into carboxylic acid by ALDH. ALDH (19 isoforms) plays a role in tissue repair and pluripotency (Balber, 2011) and across taxa (from bacteria to humans) (Yoshida et al., 1998). Various ALDH isoforms are expressed in different tissues and can metabolize different substrates. ALDH activity remained unchanged in 2D culture cells, whereas it increased in 3D cultures from 2 to 3 days after initial inoculation (Figures 2K–M). The activity of 3D-cultured cells was higher in A549 cells than in the other two normal lung cells. Activity started to increase 2 days after A549 inoculation. This result agreed with a previous report stating aldehyde degradation in A549 cells (Filipiak et al., 2010), as well as a report that shows many aldehydes decreased but alcohols increased in cancer cells (Filipiak et al., 2016). In contrast, in the case of HLB and HBEpC, the activity increase occurred 4 days after inoculation. Based on our ALDH results, the increase of the alcohol group was not related to downregulation of ALDH.

Interestingly, our ROS assay data showed that the A549 cancer cells had the lowest values of all tested cell lines. There was a decrease over time in 2D-cultured cells, whereas those in 3D cultures showed no conspicuous increase or decrease (Figures 2N–P). Although ROS must be important for VOC production, actual ROS levels inside cultured cells were not always consistent with *trans*-2-hexenol production levels in our study, which partly reflects difficulties in mimicking the tissue microenvironment (Lu et al., 2007).

In this study, we found *trans*-2-hexenol as a novel animal cellular VOC that appears to be a product of the lipid peroxidation pathway. Our study pointed to a relation solely with ADH1c upregulation. Furthermore, simple rapid sugar uptake and lactate production were not directly related to *trans*-2-hexenol increase. As such, the metabolism of *trans*-2-hexenol and its regulation still harbors some unclear points.

A *trans*-2-hexenol increase was detected in both 2D and 3D A549 cell cultures. Although the cell culture types differed somewhat (e.g., ALDH), these differences did not appear to be critical for VOC production. This study did not utilize ECM (extra cellular matrixes) or scaffold structure for 3D culture, implying that solely a 3-dimensional aggregation of cells did not significantly influence VOC production. Improving the culture techniques might uncover hidden differences between 2D and 3D.

Another remaining important point is origin of the aldehyde which would be converted into alcohol. Our data suggested the presence of *trans*-2-hexenol and implied upregulation of ADH1c, so that the *trans*-2-hexenol could be from aldehyde. Nonetheless, the synthesis of aldehyde from PUFAs (polyunsaturated fatty acids) remain unclear. There is a report that the radical species react especially with membrane PUFAs and generate lipid peroxidation products (Liou and Storz, 2010). In the case of plants, some studies show a reaction catalyzed by LOX (lipoxygenase) and HPL (Hydroperoxide lyase). This can generate several aldehydes, e.g., *trans*-2-hexenol and *cis*-3-hexenal, which are known green leaf volatiles (Hoa et al., 2015; Kunishima et al., 2016). Animal cells, in turn, possess LOX and ADH, but there is no evidence that animal cells have HPL. A reaction from PUFA to aldehyde is therefore uncertain. Two possible explanations can be forwarded. One is that radicals (ROS) non-enzymatically produced aldehyde from PUFA (Anderson and Taylor, 2012). Our ROS data, however, did not show a correlation with *trans*-2-hexenol production. Another possibility is that aldehyde, which is a precursor for *trans*-2-hexenol, is produced enzymatically, but the enzyme remains unknown. Some reports focus on enzyme promiscuity (Piedrafita et al., 2015). There are three types of promiscuities, namely substrate, catalytic, and conditional promiscuity. Enzyme conditional promiscuity can act as a reservoir of new functions, and sometimes non-canonical metabolites can be synthesized. This makes it amenable to envisage that enzyme promiscuity in cancer cells contributes to reducing the harmful effects of aldehydes or of any substances produced due to an ROS increase inside a cell.

## Data Availability Statement

The raw data supporting the conclusions of this article will be made available by the authors, without undue reservation, to any qualified researcher.

## Author Contributions

TF conducted GC-MS analysis and designed all experiments and wrote paper. HO assisted VOC analysis by GC-MS and enzyme assay. RI cultured all cells in this study and conducted qPCR. SO contributed to develop VOC procedure. All authors contributed to the article and approved the submitted version.

## Conflict of Interest

TF, RI, and HO were employed by the company Anicom Specialty Medicinal Institute. SO was employed by the company GL Sciences Inc.

## Supplementary Material

Figure S1 Experimental scheme to capture VOC from cell culture flasks. For cellular VOC sampling, monotrap was placed into a 2.5 mL syringe connected to a cell culture flask cap via a connector. Pumping was conducted at constant speed for 30 min, which corresponds to ~750 pumping actions.

Figure S2 12 VOCs (acetate, benzaldehyde, benzyl alcohol, butyrate, caproate, 1-decanol, decanal, dodecanal, 2-ethyl-1-hexanol, formate, heptadecane, heptanoate) profiles by GC-MS. Y axis indicates extracted ion counting (EIC) of each VOC. sampling was done at 0, 3, and 4 days after inoculation (i.e., D0, D3, and D4). Experiments were biologically triplicated. Error bars: standard deviation. Medium (cell-less media), HLB (Human lung fibroblasts cells), HBEpC (Human bronchial epithelial cells as primary cells), and A549 (human lung adenocarcinoma) were compared in both 2D and 3D culture.

Figure S3 12 VOC (hexadecane, 4-hydroxy-nonenal, isobutyrate, 3-methyl pentanoate, nonanal, 1- nonanol, octadecane, pentadecane, phenol, propionate, toluene, tetradecane) profiles by GC-MS. Y axis indicates extracted ion counting (EIC) of each VOC. Sampling was done at 0, 3, and 4 days after inoculation (i.e., D0, D3, and D4). Experiments were biologically triplicated. Error bars: standard deviation. Medium (cell-less media), HLB (Human lung fibroblasts cells), HBEpC (Human bronchial epithelial cells as primary cells), and A549 (human lung adenocarcinoma) were compared in both 2D and 3D culture.

Figure S4 6 VOC (*trans*-2-hexenol, tridecane, undecane, undecanal, 1-undecanol, valerate) profiles by GC-MS. Y axis indicates extracted ion counting (EIC) of each VOC. Sampling was done at 0, 3, and 4 days after inoculation (i.e., D0, D3, and D4). Experiments were biologically triplicated. Error bars: standard deviation. Medium (cell-less media), HLB (Human lung fibroblasts cells), HBEpC (Human bronchial epithelial cells as primary cells), and A549 (human lung adenocarcinoma) were compared both 2D and 3D culture.

Figure S5 Calibration curve of VOCs. **(A)** tetradecane (20–1,000 nmole), **(B)** *trans*-2-hexenol (5–500 nmole), and **(C)** isobutyrate (50–5,000 nmole). Reference compounds was added into T75 culture flask (270 mL capacity) with 10 mL water, and volatilized compounds in a flask were trapped by monotrap (RG and RSC18) in a 2.5 mL syringe, the same as used in the enrichment of cellular VOCs (30 min pumping) and measured by GC-MS. VOCs were enriched after sample preparation. X axis indicates nmole reference compounds in 10 mL. Y axis indicates extracted ion counting (EIC) of each VOC. Experiments were triplicated. Error bars: standard deviation.

Figure S6 qPCR data of ALR2 (Aldehyde reductase) and FAR1 (Fatty acyl-CoA reductase). A549 (human lung adenocarcinoma), and HBEpC (Human bronchial epithelial cells as primary cells) were compared. Sampling was done at 1, 2, 3, 4 days after inoculation. Experiments were biologically triplicated. Y axis indicates relative gene expression to GAPDH. Error bars: standard deviation.

Figure S7 Metabolic pathway [modified from (Ruffing, 2013)]. Fatty alcohol can be generated either from aldehyde or fatty acyl-CoA (R-SCoA). Fatty aldehyde can be derived from lipid peroxidation or from alcohol (Alcohol dehydrogenase; ADH/Aldehyde reductase; ALR). Compared with single-cell organisms, it is unclear whether metazoans possess enzymes to convert R-SCoA into aldehyde (Acyl ACP reductase; AAR), or aldehyde into hydrocarbon (Aldehyde decarbonylase; ADC).

Table S1 Metabolite list. Total 30 VOC reference compounds for GC-MS profiling with retention time, chosen fragment *m/z* as well as CAS number.

Table S2 Primer sequences used in qPCR. F and R indicates forward and reverse primers respectively.

Table S3 VOC list of p-value between Days 0 and 4 (Cell less media, A549, HLB, and HBEpC). VOC whose *p*-value was below 0.05 were listed here. Red and blue letter indicate increase and decrease from Day 0, respectively. 2D and 3D indicates 2 dimensional and 3 dimensional culture respectively.

Table S4 VOC list of p-value between cell less media (Day 4) and cell culture (A549, HLB, and HBEpC) (Day 4). VOC whose *p*-value was below 0.05 were listed here. Red and blue letter indicate increase and decrease to cell-less media respectively. In all cell samples, benzaldehyde decrease to media was common characteristics.

## Abbreviations

A549: Human lung adenocarcinoma
AAR: Acyl ACP reductase
ADC: Aldehyde decarbonylase
ADH: Alcohol dehydrogenase
ALA: Alpha linoleic acid
ALDH: Aldehyde dehydrogenase
ALR: Aldehyde reductase
ATP: Adenosine triphosphate
DMEM: Dulbecco's modified eagle medium
ECM: Extra cellular matrixes
EIC: Extracted ion counting
EI-MS: Electron ionization
FAR: Fatty acyl-CoA reductase
FCS: Fetal bovine serum
GC: Gas chromatography
GAPDH: Glyceraldehyde-3-phosphate dehydrogenase
HBEpC: Human bronchial epithelial cells as primary cells
HLB: Human lung fibroblasts cells
HPL: Hydroperoxide lyase
IR: Infrared spectroscopy
ITEX: In tube extraction
LOX: Lipoxygenase
MS: Mass spectrometry
NAD: Nicotinamide adenine dinucleotide
NTME: Needle trap extraction
PBS: Phosphate-buffered saline
PCA: Principle components analysis
PTR: Proton transfer reaction
PUFA: Polyunsaturated fatty acid
qPCR: Quantitative polymerase chain reaction
ROS: Reactive oxygen species
SCFA: Short chain fatty acid
SIFT: Selected ion flow tube
SPME: Solid phase micro extraction
TD: Thermal desorption
VOC: Volatile organic compounds.
