# Supplementary figures and images for: Elucidation of Biochemical Pathways Underlying VOCs Production in A549 Cells
Source: Front Mol Biosci. 2020 Jun 30;7:116. doi: 10.3389/fmolb.2020.00116 (PMC7338772; doi:10.3389/fmolb.2020.00116)

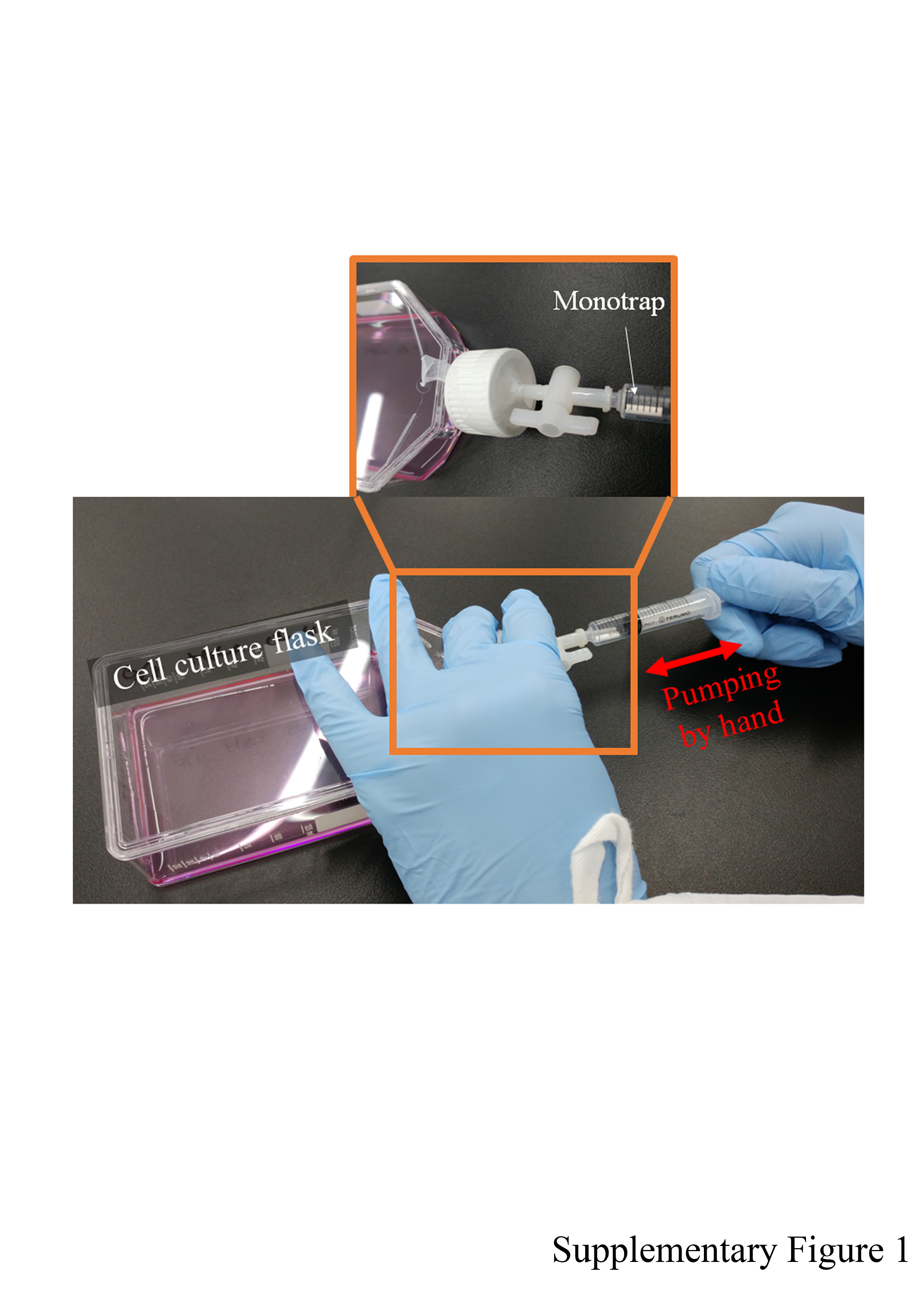

Supplement: Figure S1 — Experimental scheme to capture VOC from cell culture flasks. For cellular VOC sampling, monotrap was placed into a 2.5 mL syringe connected to a cell culture flask cap via a connector. Pumping was conducted at constant speed for 30 min, which corresponds to ~750 pumping actions. [file Supplementary_Figure_1.TIF]

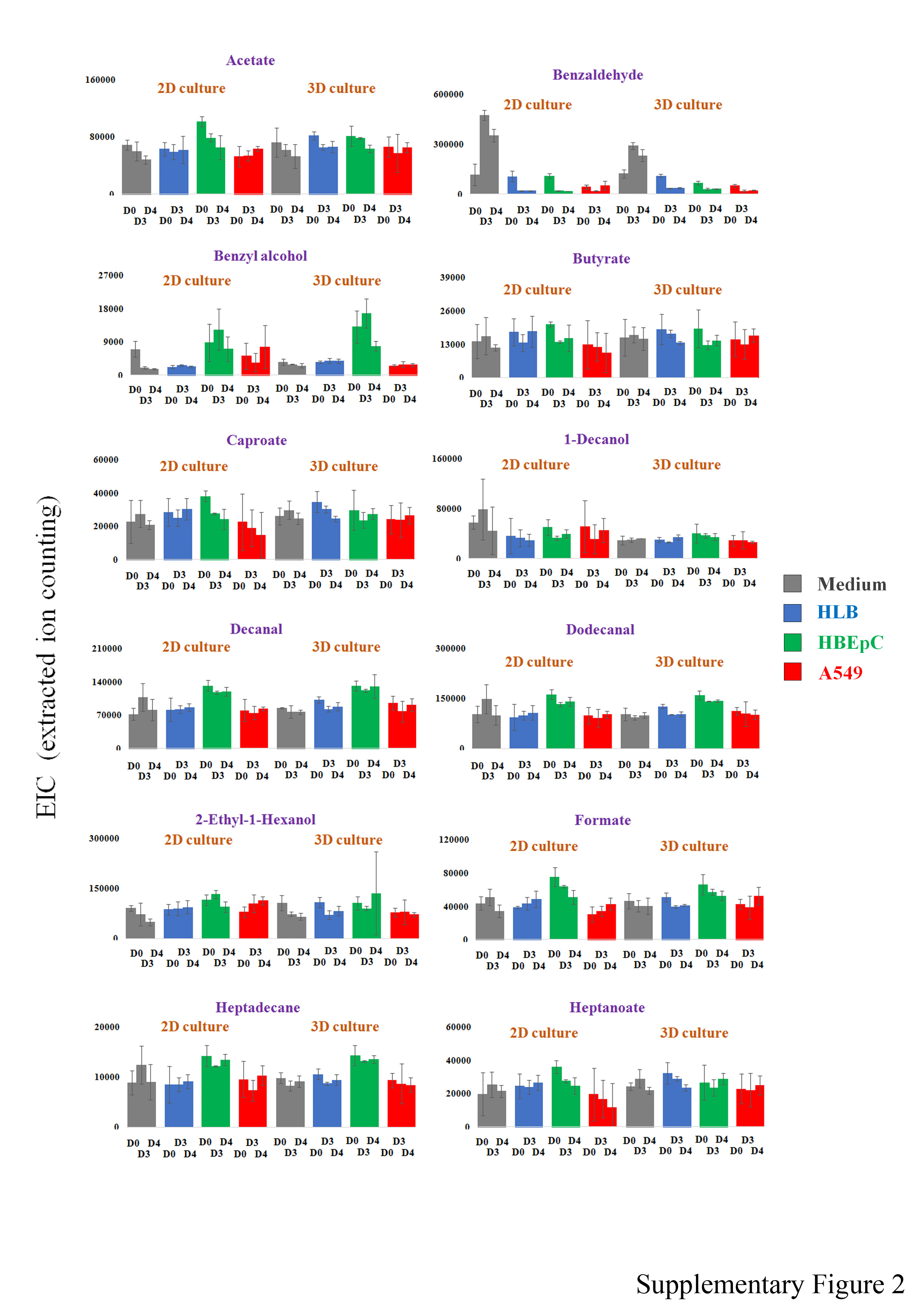

Supplement: Figure S2 — 12 VOCs (acetate, benzaldehyde, benzyl alcohol, butyrate, caproate, 1-decanol, decanal, dodecanal, 2-ethyl-1-hexanol, formate, heptadecane, heptanoate) profiles by GC-MS. Y axis indicates extracted ion counting (EIC) of each VOC. sampling was done at 0, 3, and 4 days after inoculation (i.e., D0, D3, and D4). Experiments were biologically triplicated. Error bars: standard deviation. Medium (cell-less media), HLB (Human lung fibroblasts cells), HBEpC (Human bronchial epithelial cells as primary cells), and A549 (human lung adenocarcinoma) were compared in both 2D and 3D culture. [file Supplementary_Figure_2.TIF]

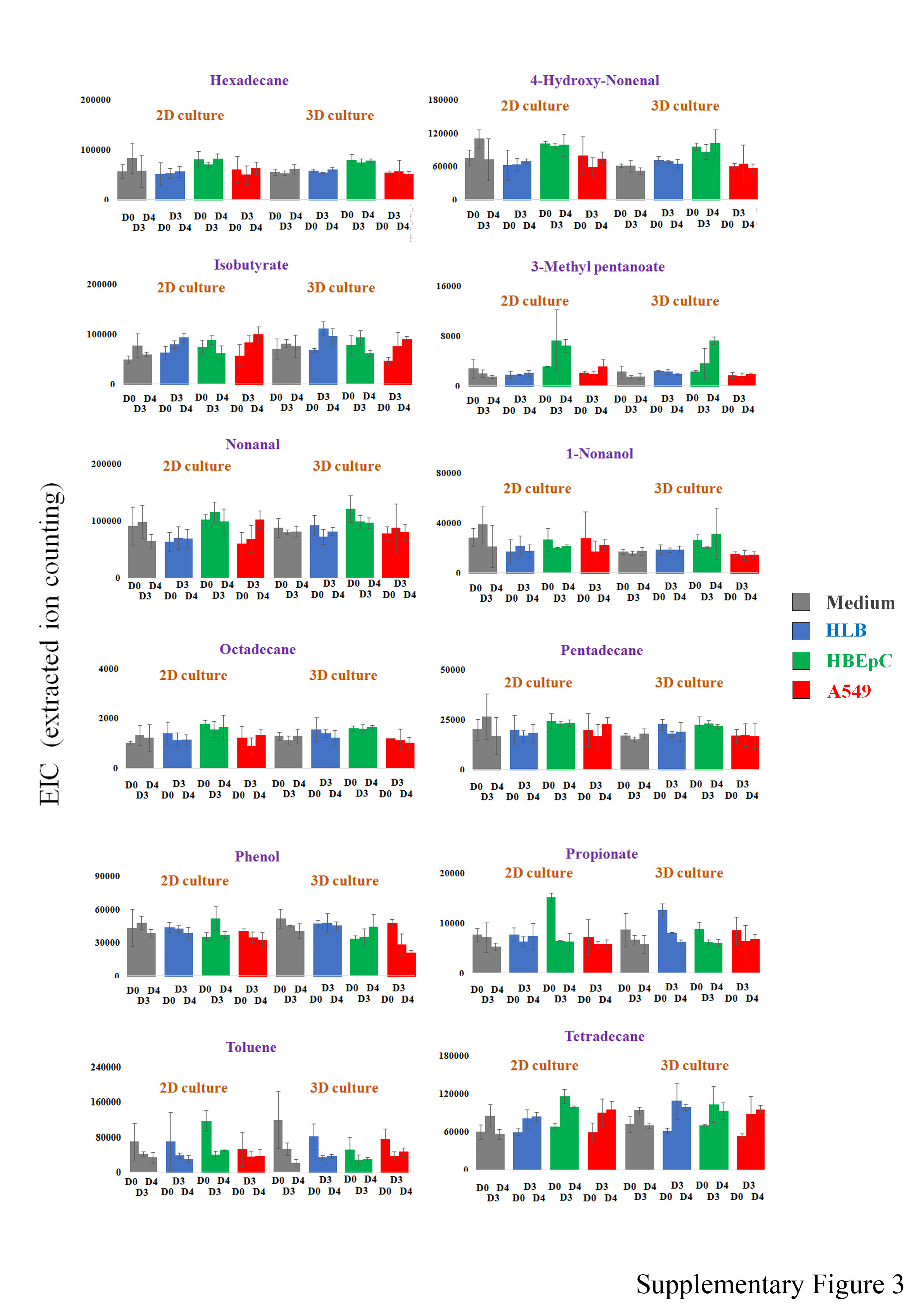

Supplement: Figure S3 — 12 VOC (hexadecane, 4-hydroxy-nonenal, isobutyrate, 3-methyl pentanoate, nonanal, 1- nonanol, octadecane, pentadecane, phenol, propionate, toluene, tetradecane) profiles by GC-MS. Y axis indicates extracted ion counting (EIC) of each VOC. Sampling was done at 0, 3, and 4 days after inoculation (i.e., D0, D3, and D4). Experiments were biologically triplicated. Error bars: standard deviation. Medium (cell-less media), HLB (Human lung fibroblasts cells), HBEpC (Human bronchial epithelial cells as primary cells), and A549 (human lung adenocarcinoma) were compared in both 2D and 3D culture. [file Supplementary_Figure_3.TIFF]

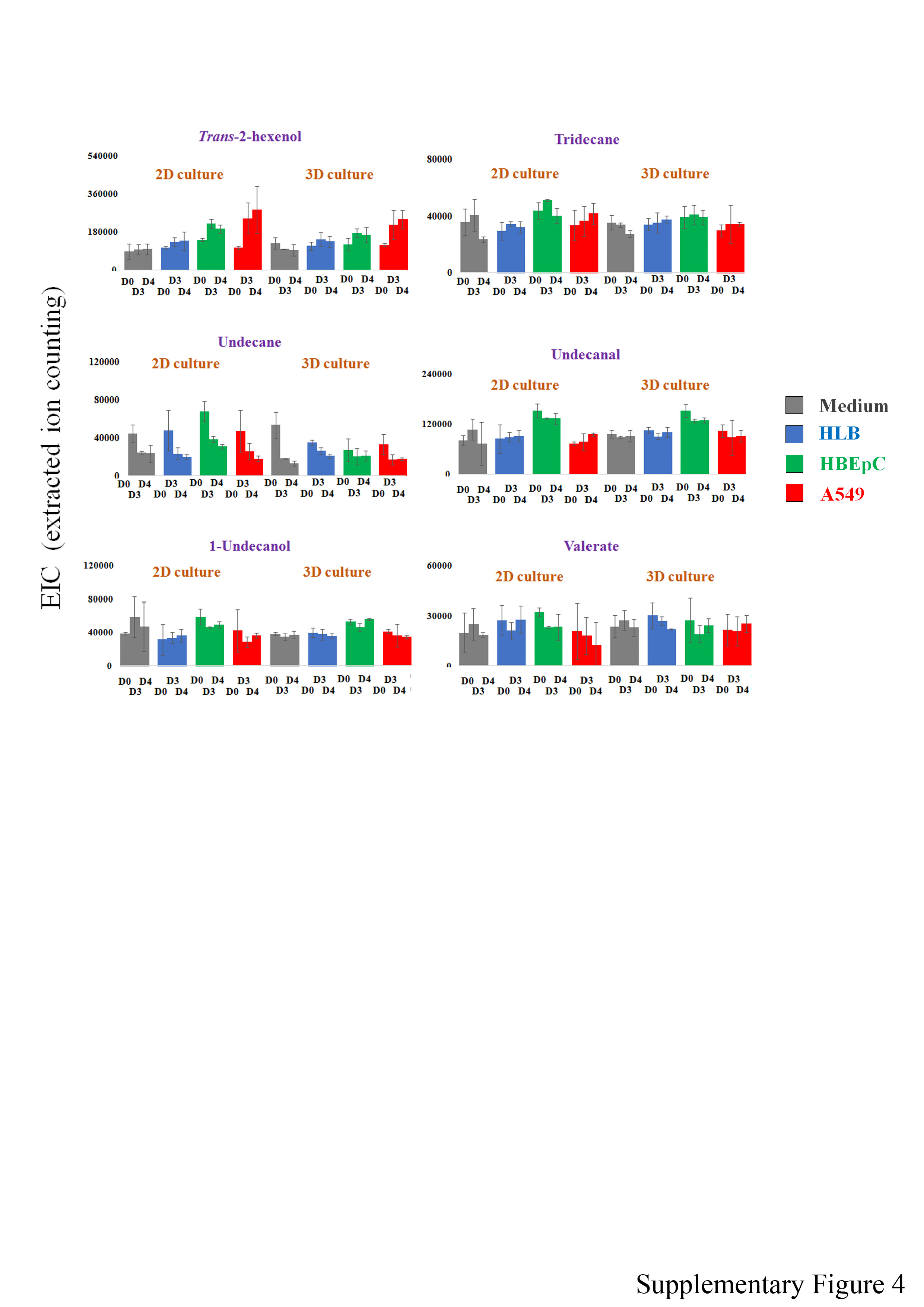

Supplement: Figure S4 — 6 VOC (trans-2-hexenol, tridecane, undecane, undecanal, 1-undecanol, valerate) profiles by GC-MS. Y axis indicates extracted ion counting (EIC) of each VOC. Sampling was done at 0, 3, and 4 days after inoculation (i.e., D0, D3, and D4). Experiments were biologically triplicated. Error bars: standard deviation. Medium (cell-less media), HLB (Human lung fibroblasts cells), HBEpC (Human bronchial epithelial cells as primary cells), and A549 (human lung adenocarcinoma) were compared both 2D and 3D culture. [file Supplementary_Figure_4.TIF]

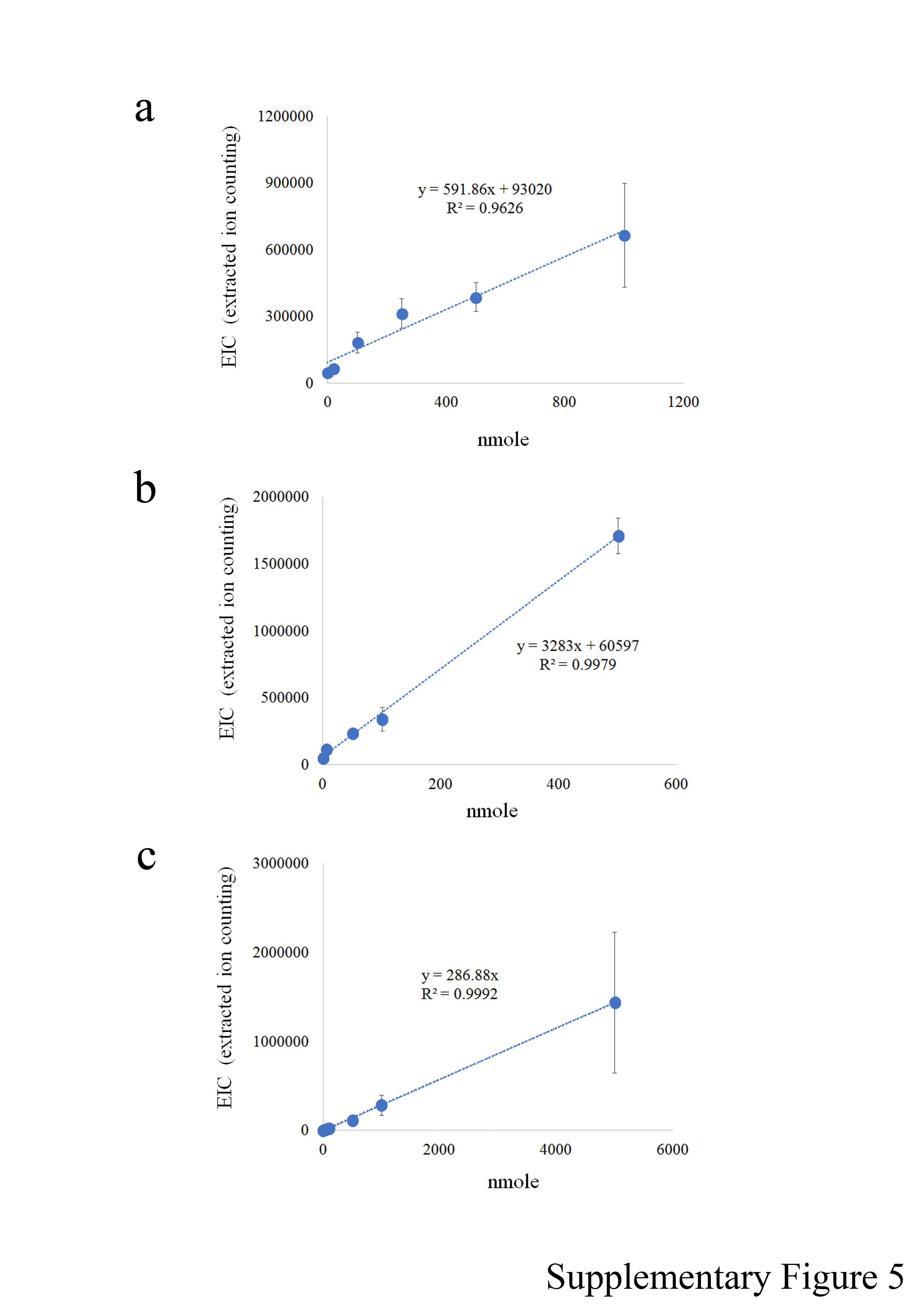

Supplement: Figure S5 — Calibration curve of VOCs. (A) tetradecane (20–1,000 nmole), (B) trans-2-hexenol (5–500 nmole), and (C) isobutyrate (50–5,000 nmole). Reference compounds was added into T75 culture flask (270 mL capacity) with 10 mL water, and volatilized compounds in a flask were trapped by monotrap (RG and RSC18) in a 2.5 mL syringe, the same as used in the enrichment of cellular VOCs (30 min pumping) and measured by GC-MS. VOCs were enriched after sample preparation. X axis indicates nmole reference compounds in 10 mL. Y axis indicates extracted ion counting (EIC) of each VOC. Experiments were triplicated. Error bars: standard deviation. [file Supplementary_Figure_5.TIF]

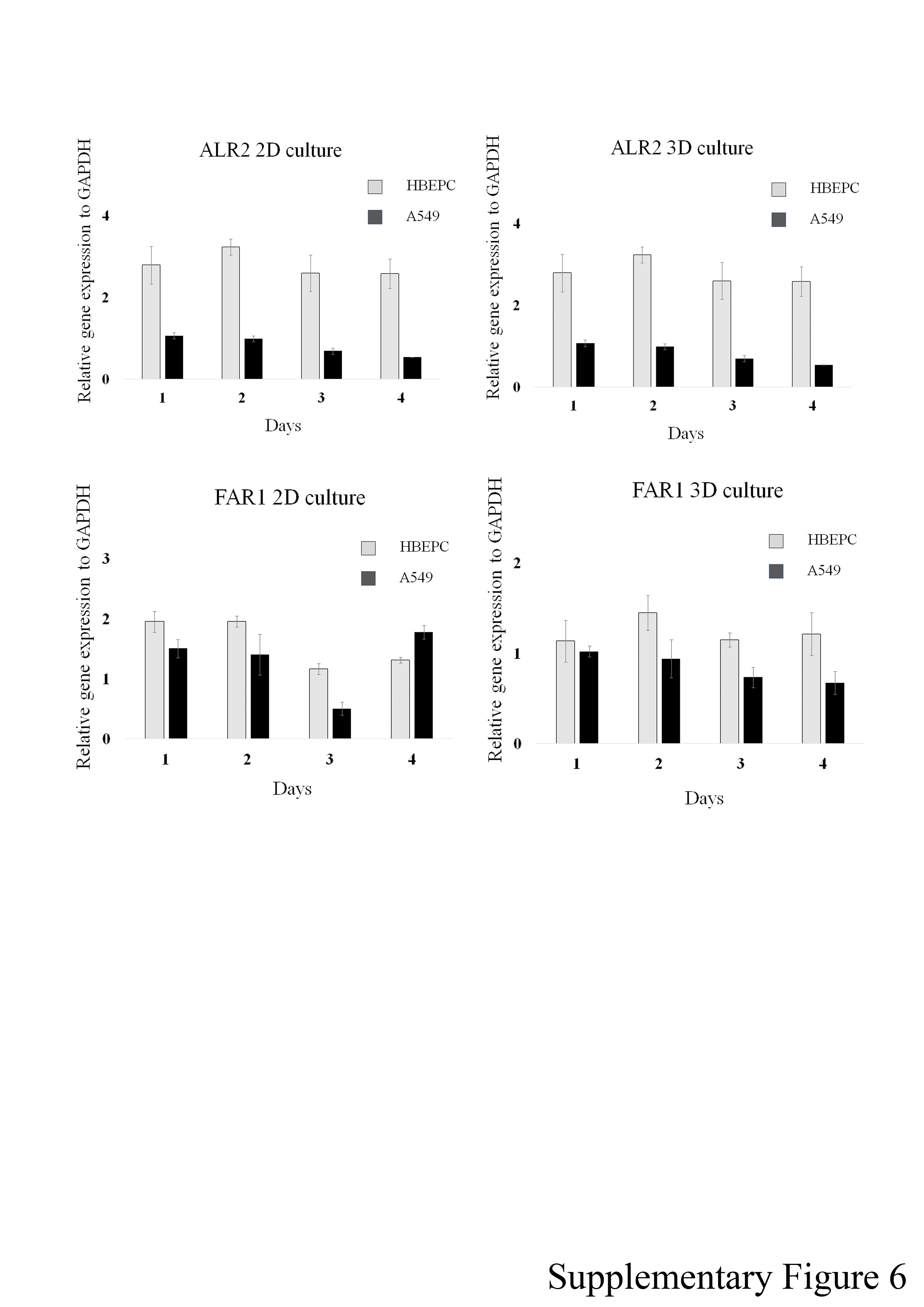

Supplement: Figure S6 — qPCR data of ALR2 (Aldehyde reductase) and FAR1 (Fatty acyl-CoA reductase). A549 (human lung adenocarcinoma), and HBEpC (Human bronchial epithelial cells as primary cells) were compared. Sampling was done at 1, 2, 3, 4 days after inoculation. Experiments were biologically triplicated. Y axis indicates relative gene expression to GAPDH. Error bars: standard deviation. [file Supplementary_Figure_6.TIF]

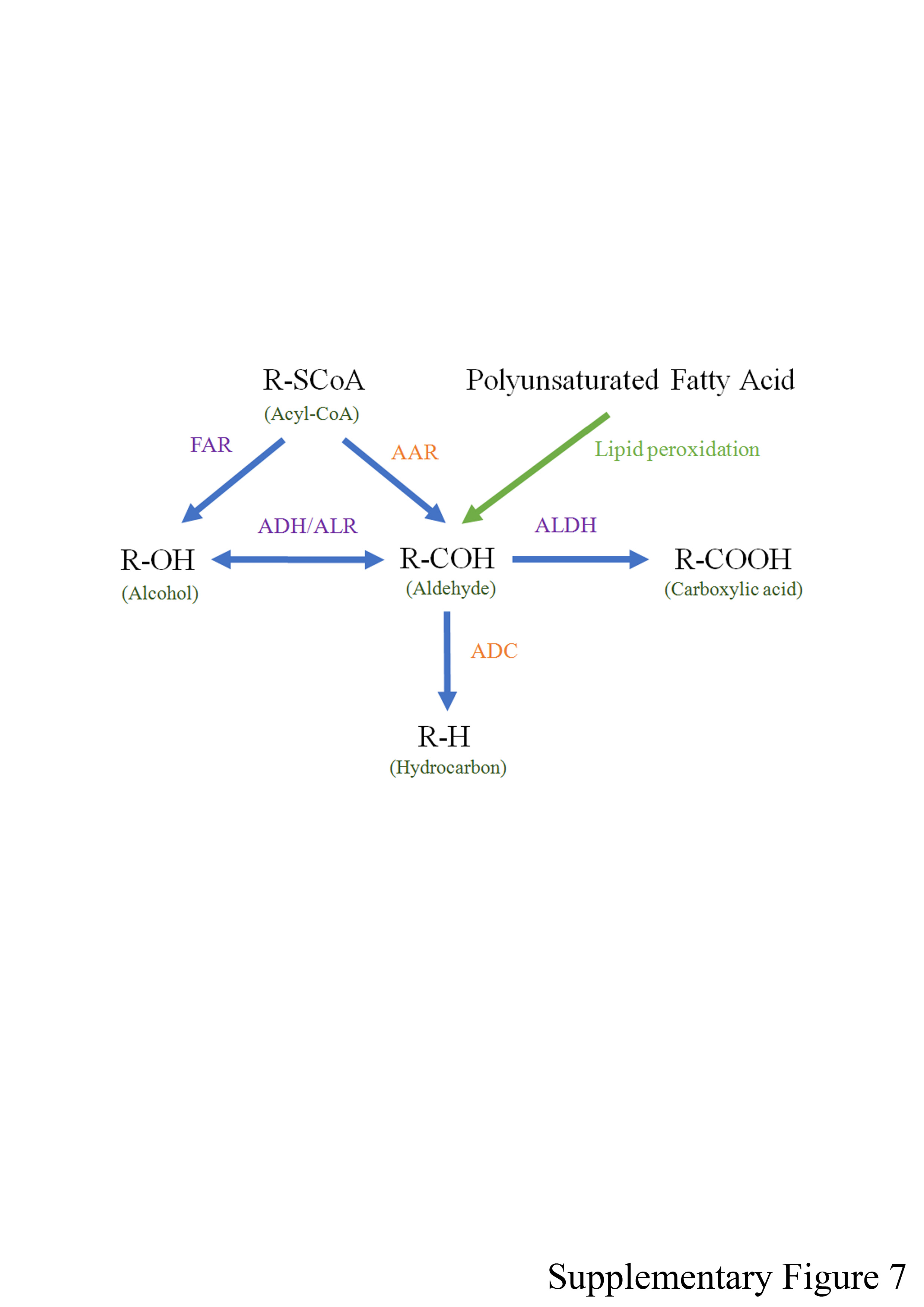

Supplement: Figure S7 — Metabolic pathway [modified from (Ruffing, 2013)]. Fatty alcohol can be generated either from aldehyde or fatty acyl-CoA (R-SCoA). Fatty aldehyde can be derived from lipid peroxidation or from alcohol (Alcohol dehydrogenase; ADH/Aldehyde reductase; ALR). Compared with single-cell organisms, it is unclear whether metazoans possess enzymes to convert R-SCoA into aldehyde (Acyl ACP reductase; AAR), or aldehyde into hydrocarbon (Aldehyde decarbonylase; ADC). [file Supplementary_Figure_7.TIF]
