# Supplementary material for: Elucidation of Biochemical Pathways Underlying VOCs Production in A549 Cells
Source: Front Mol Biosci. 2020 Jun 30;7:116. doi: 10.3389/fmolb.2020.00116 (PMC7338772; doi:10.3389/fmolb.2020.00116)
Supplement: Table S1 — Metabolite list. Total 30 VOC reference compounds for GC-MS profiling with retention time, chosen fragment m/z as well as CAS number. [file Table_1.pdf]

Supplementary Table 1 Metabolite list. Total 30 VOC reference compounds for GC-MS profiling with retention time, chosen fragment *m/z* as well as CAS number.

Name of metabolite	Retention time	*m/z*	CAS
Toluene	3.88	91	108-88-3
Undecane	5.10	156	1120-21-4
Tridecane	10.60	184	629-50-5
Nonanal	13.50	98	124-19-6
Tetradecane	13.90	198	629-59-4
trans 2-hexenol	13.93	82	928-95-0
Acetate	14.79	60	64-19-7
Formate	15.98	46	64-18-6
2-Ethyl-1-Hexanol	16.35	83	104-76-7
Decanal	16.50	82	112-31-2
Benzaldehyde	16.58	106	100-52-7
Pentadecane	16.80	212	629-62-9
Propionate	17.13	74	79-09-4
Isobutyrate	17.99	73	79-31-2
Undecanal	19.00	82	112-44-7
Butyrate	19.42	60	107-92-6
Hexadecane	19.50	226	544-76-3
1-Nonanol	20.50	98	143-08-8
Dodecanal	21.60	82	112-54-9
Valerate	21.99	60	109-52-4
Heptadecane	22.00	240	629-78-7
1-Decanol	22.90	112	112-30-1
3-methyl pentanoate	23.27	60	105-43-1
Octadecane	24.20	254	593-45-3
Caproate	24.40	60	142-62-1
Benzyl alcohol	24.50	108	100-51-6
1-Undecanol	25.10	126	112-42-5
Heptanoate	26.10	60	111-14-8
Phenol	26.20	94	108-95-2
4-Hydroxy-Nonenal	27.70	57	75899-68-2
