# Supplementary material for: Elucidation of Biochemical Pathways Underlying VOCs Production in A549 Cells
Source: Front Mol Biosci. 2020 Jun 30;7:116. doi: 10.3389/fmolb.2020.00116 (PMC7338772; doi:10.3389/fmolb.2020.00116)
Supplement: Table S2 — Primer sequences used in qPCR. F and R indicates forward and reverse primers respectively. [file Table_2.pdf]

Supplementary Table 2 Primer sequences used in qPCR. F and R indicates forward and reverse primers respectively.

Gene name	Primer sequence
ADH1c-F	CTCGCCCCTGGAGAAAGTC
ADH1c-R	GGCCCCCAACTCTTTAGCC
FAR1-F	AGACACCACAAGAGCGAGTG
FAR1-R	CCAGTTTAGGTTGGGTGAGTTC
ALR2-F	TTTTCCCATTGGATGAGTCGG
ALR2-R	CCTGGAGATGGTTGAAGTTGG
