# Supplementary material for: Elucidation of Biochemical Pathways Underlying VOCs Production in A549 Cells
Source: Front Mol Biosci. 2020 Jun 30;7:116. doi: 10.3389/fmolb.2020.00116 (PMC7338772; doi:10.3389/fmolb.2020.00116)
Supplement: Table S3 — VOC list of p-value between Days 0 and 4 (Cell less media, A549, HLB, and HBEpC). VOC whose p-value was below 0.05 were listed here. Red and blue letter indicate increase and decrease from Day 0, respectively. 2D and 3D indicates 2 dimensional and 3 dimensional culture respectively. [file Table_3.pdf]

Supplementary Table 3 VOC list of *p*-value between Day0 and Day4 (Cell less media, A549, HLB and HBEpC). VOC whose *p*-value was below 0.05 were listed here. Red and blue letter indicate increase and decrease from Day0 respectively. 2D and 3D indicates 2dimensional and 3dimensional culture respectively.

	Medium_2D		A549_2D		HLB_2D		HBEpC_2D
2-Ethyl-1-Hexanol	0.004	Undecanal	0.003	Tetradecane	0.01	Tetradecane	0.0004
Benzaldehyde	0.005	Tetradecane	0.03	Benzaldehyde	0.01	Benzaldehyde	0.0005
Benzyl alcohol	0.01	Nonanal	0.04	Isobutyrate	0.02	Propionate	0.001
Acetate	0.01	2-Ethyl-1-Hexanol	0.04			Undecane	0.004
Propionate	0.04	Trans2-hexanol	0.04			3-Methyl pentanoate	0.006
Undecane	0.04					Toluene	0.008
						Trans2-hexenol	0.01
						Caproate	0.02
						Acetate	0.02
						Heptanoate	0.03
						Formate	0.04

	Medium_3D		A549_3D		HLB_3D		HBEpC_3D
Undecane	0.007	Phenol	0.0002	Benzaldehyde	0.0004	3-Methyl pentanoate	0.0001
Benzaldehyde	0.01	Tetradecane	0.0007	Tetradecane	0.0004	Benzaldehyde	0.005
Decanal	0.04	Isobutyrate	0.001	Propionate	0.001	Tetradecane	0.03
2-Ethyl-1-Hexanol	0.04	Benzaldehyde	0.005	Undecane	0.001	Propionate	0.03
		Trans2-hexenol	0.009	3-methyl pentanoate	0.004		
		1-Undecanol	0.01				
