# Supplementary material for: Elucidation of Biochemical Pathways Underlying VOCs Production in A549 Cells
Source: Front Mol Biosci. 2020 Jun 30;7:116. doi: 10.3389/fmolb.2020.00116 (PMC7338772; doi:10.3389/fmolb.2020.00116)
Supplement: Table S4 — VOC list of p-value between cell less media (Day 4) and cell culture (A549, HLB, and HBEpC) (Day 4). VOC whose p-value was below 0.05 were listed here. Red and blue letter indicate increase and decrease to cell-less media respectively. In all cell samples, benzaldehyde decrease to media was common characteristics. [file Table_4.pdf]

Supplementary Table 4 VOC list of *p*-value between cell less media (Day4) and cell culture (A549, HLB and HBEpC) (Day4). VOC whose *p*-value was below 0.05 were listed here. Red and blue letter indicate increase and decrease to cell-less media respectively. In all cell samples, benzaldehyde decrease to media was common characteristics.

	2D culture A549 (vs Media)		2D culture HLB (vs Media)		2D culture HBEpC (vs Media)
Benzaldehyde	0.0003	Benzaldehyde	0.0001	Benzaldehyde	0.00009
2-Ethyl-1-Hexanol	0.001	Isobutyrate	0.005	Tetradecane	0.0007
Tetradecane	0.01	Tetradecane	0.01	3-Methyl pentanoate	0.001
Isobutyrate	0.01	Benzyl alcohol	0.01	Trans2-hexenol	0.006
Tridecane	0.01	2-Ethyl-1-Hexanol	0.02	Tridecane	0.007
Acetate	0.01	Tridecane	0.03	2-Ethyl-1-Hexanol	0.01
Nonanal	0.02			Benzyl alcohol	0.04
Trans2-hexenol	0.04				

	3D culture A549 (vs Media)		3D culture HLB (vs Media)		3D culture HBEpC (vs Media)
Benzaldehyde	0.0005	Benzaldehyde	0.0008	3-Methyl pentanoate	0.0001
Tetradecane	0.004	Tetradecane	0.001	Benzaldehyde	0.0007
Phenol	0.007	Tridecane	0.005	Dodecanal	0.001
1-Decanol	0.008	Undecane	0.01	1-Undecanol	0.001
Trans2-hexenol	0.008			Benzyl alcohol	0.003
Tridecane	0.01			Heptadecane	0.005
Toluene	0.02			Undecanal	0.01
Undecane	0.03			4-Hydroxy-Nonenal	0.02
				Tridecane	0.02
				Decanal	0.02
				Heptanoate	0.03
				Tetradecane	0.03
